# Supplementary material for: Sensitive and error-tolerant annotation of protein-coding DNA with BATH
Source: bioRxiv. 2024 Jan 1:2023.12.31.573773. Preprint. [Version 1] doi: 10.1101/2023.12.31.573773 (PMC10802276; doi:10.1101/2023.12.31.573773)
Supplement: 1 [file NIHPP2023.12.31.573773v1-supplement-1.pdf]

bioRxiv preprint doi: https://doi.org/10.1101/2023.12.31.573773; this version posted January 1, 2024. The copyright holder for this preprint (which was not certified by peer review) is the author/funder, who has granted bioRxiv a license to display the preprint in perpetuity. It is made available under aCC-BY 4.0 International license.

Supplementary Materials

Additional supplementary materials can be found at https://github.com/TravisWheelerLab/BATH-paper, including code for construction and analysis of *trasnsmark* benchmarks.

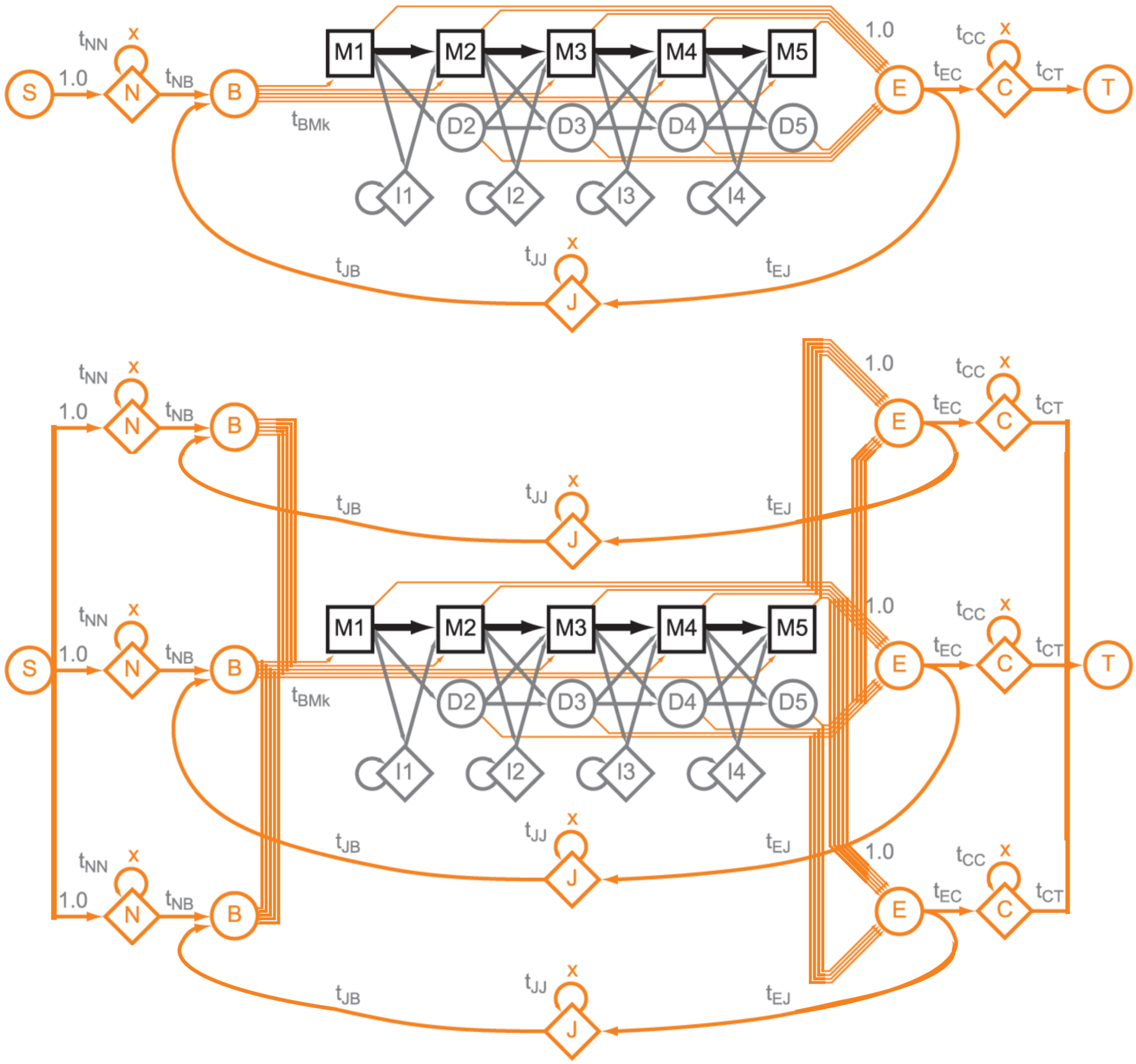

Fig. S1. Illustration of the pHMM "special states" (shown in orange) in the protein pHMM used by HMMER3 (14) and BATH and the frameshift-aware codon pHMM used by BATH. These states allow for local alignments by permitting any non-homologous regions of the target to align to these special states rather than to the core model. The FA codon model uses three sets of the N, B, J, E, and C states, one for each translation frame. This forces the model to address any frameshifts inside the core model by emitting a quasi-codon, rather than in the special states.

bioRxiv preprint doi: https://doi.org/10.1101/2023.12.31.573773; this version posted January 1, 2024. The copyright holder for this preprint (which was not certified by peer review) is the author/funder, who has granted bioRxiv a license to display the preprint in perpetuity. It is made available under aCC-BY 4.0 International license.

/* Special conditions for 0-4th, L^{th} and K^{th} indices are omitted for simplicity */

$L \leftarrow$ length of target DNA window

$K \leftarrow$ length of FA codon model

$Memo[4][K]$; // Matrix for storing memoized values

$M[L][K][6]$; // Match state matrix

$I[L][K]$; // Insert state matrix

$D[L][K]$; // Delete state matrix

/* $T(X,Y)$ is the log transition probability from state X to state Y */

/* $E_V(M_j(x_i))$ is the log emissions probability from the jth M state of a (pseudo-)codon of length V ending with the ith target nucleotide */

for *i in {1..L}* **do**

  $N(i) \leftarrow N(i-3) + T(N,N)$; // N state transitions to N state

  $B(i) \leftarrow log_2\left(\sum\left\{\begin{array}{l} 2^{N(i)+T(N,B)} \\ 2^{J(i)+T(J,B)} \end{array}\right\}\right)$; // N state transitions to B state // J state transitions to B state

  for *j in {1..K}* **do**

    for *m in {4..1}* **do**

      $Memo(m+1,j) \leftarrow Memo(m,j)$; // Shift memoized values up by one row

    end

    $Memo(1,j) \leftarrow log_2\left(\sum\left\{\begin{array}{l} 2^{B(i-1)+T(B,M)} \\ 2^{M(i-1,j-1)+T(M_{j-1},M_j)} \\ 2^{I(i-1,j-1)+T(I_{j-1},M_j)} \\ 2^{D(i-1,j-1)+T(D_{j-1},M_j)} \end{array}\right\}\right)$; // B state transitions to M state // M state transitions to M state // I state transitions to M state // D state transitions to M state

    $M(i,j,1) \leftarrow Memo(1,j) + E_1(M_j(x_i))$; // M state emits length 1 pseudo-codon

    $M(i,j,2) \leftarrow Memo(2,j) + E_2(M_j(x_i))$; // M state emits length 2 pseudo-codon

    $M(i,j,3) \leftarrow Memo(3,j) + E_3(M_j(x_i))$; // M state emits codon

    $M(i,j,4) \leftarrow Memo(4,j) + E_4(M_j(x_i))$; // M state emits length 4 pseudo-codon

    $M(i,j,5) \leftarrow Memo(5,j) + E_5(M_j(x_i))$; // M state emits length 5 pseudo-codon

    $M(i,j,0) \leftarrow log_2(2^{M(i,j,1)} + 2^{M(i,j,2)} + 2^{M(i,j,3)} + 2^{M(i,j,4)} + 2^{M(i,j,5)})$; // Sum all M state emissions

    $I(i,j) \leftarrow log_2\left(\sum\left\{\begin{array}{l} 2^{M(i-3,j)+T(M_j,I_j)} \\ 2^{I(i-3,j)+T(I_j,I_j)} \end{array}\right\}\right)$; // M state transitions to I state // I state transitions to I state

    $D(i,j) \leftarrow log_2\left(\sum\left\{\begin{array}{l} 2^{M(i,j-1)+T(M_{j-1},D_j)} \\ 2^{D(i,j-1)+T(D_{j-1},D_j)} \end{array}\right\}\right)$; // M state transitions to D state // D state transitions to D state

    $E(i) \leftarrow log_2\left(\sum\left\{\begin{array}{l} 2^{M(i,j)} \\ 2^{D(i,j)} \\ 2^{E(i)} \end{array}\right\}\right)$; // M state transitions to D state // D state transitions to E state // Sum with all transitions to E state

  end

  $J(i) \leftarrow log_2\left(\sum\left\{\begin{array}{l} 2^{E(i)+T(E,J)} \\ 2^{J(i-3)+T(J,J)} \end{array}\right\}\right)$; // E state transitions to J state // J state transitions to J state

  $C(i) \leftarrow log_2\left(\sum\left\{\begin{array}{l} 2^{E(i)+T(E,C)} \\ 2^{C(i-3)+T(J,C)} \end{array}\right\}\right)$; // E state transitions to C state // C state transitions to C state

end

$T \leftarrow log_2\left(\sum\left\{\begin{array}{l} 2^{C(L-2)} \\ 2^{C(L-1)} \\ 2^{C(L)} \end{array}\right\}\right) + T(C,T)$; // C state transitions to T state in all three frames

Fig. S2. Pseudocode for the frameshift-aware Forward filter algorithm used by bathsearch. The FA algorithms employed by bathsearch use larger matrices, both because DNA targets have three times the residues of their protein counterparts and because the probabilities of various (quasi-)codon lengths often need to be stored separately, quintupling the number of M state cells. They also require more operations at each i,j position (where i is a residue in the target and j is a position in the model) to account for each of the separate (quasi-)codon emission probabilities and transition lookbacks. FA matrices also resist the application of the SIMD optimizations employed by HMMER3 for protein-to-protein alignment. In a protein-to-protein matrix such as (A), each new cell in row i can only transition from a cell in row i or row i-1. In (B), however, a cell in row i can transition from a cell in row i, i-1, 1-2, i-3, i-4, or i-5. This prevents straightforward use of the "sparse rescaling" employed in HMMER3 to prevent underflow in SIMD calculations (14). Therefore all FA algorithms in BATH are implemented without the benefit of SIMD optimization. To avoid repeated calculations, a "Memo" matrix is used to store values that will be used by subsequent M(i,j) cells. This dramatically reduces the number of additional calculations needed for frameshift-aware Forward versus standard Forward.

bioRxiv preprint doi: https://doi.org/10.1101/2023.12.31.573773; this version posted January 1, 2024. The copyright holder for this preprint (which was not certified by peer review) is the author/funder, who has granted bioRxiv a license to display the preprint in perpetuity. It is made available under aCC-BY 4.0 International license.

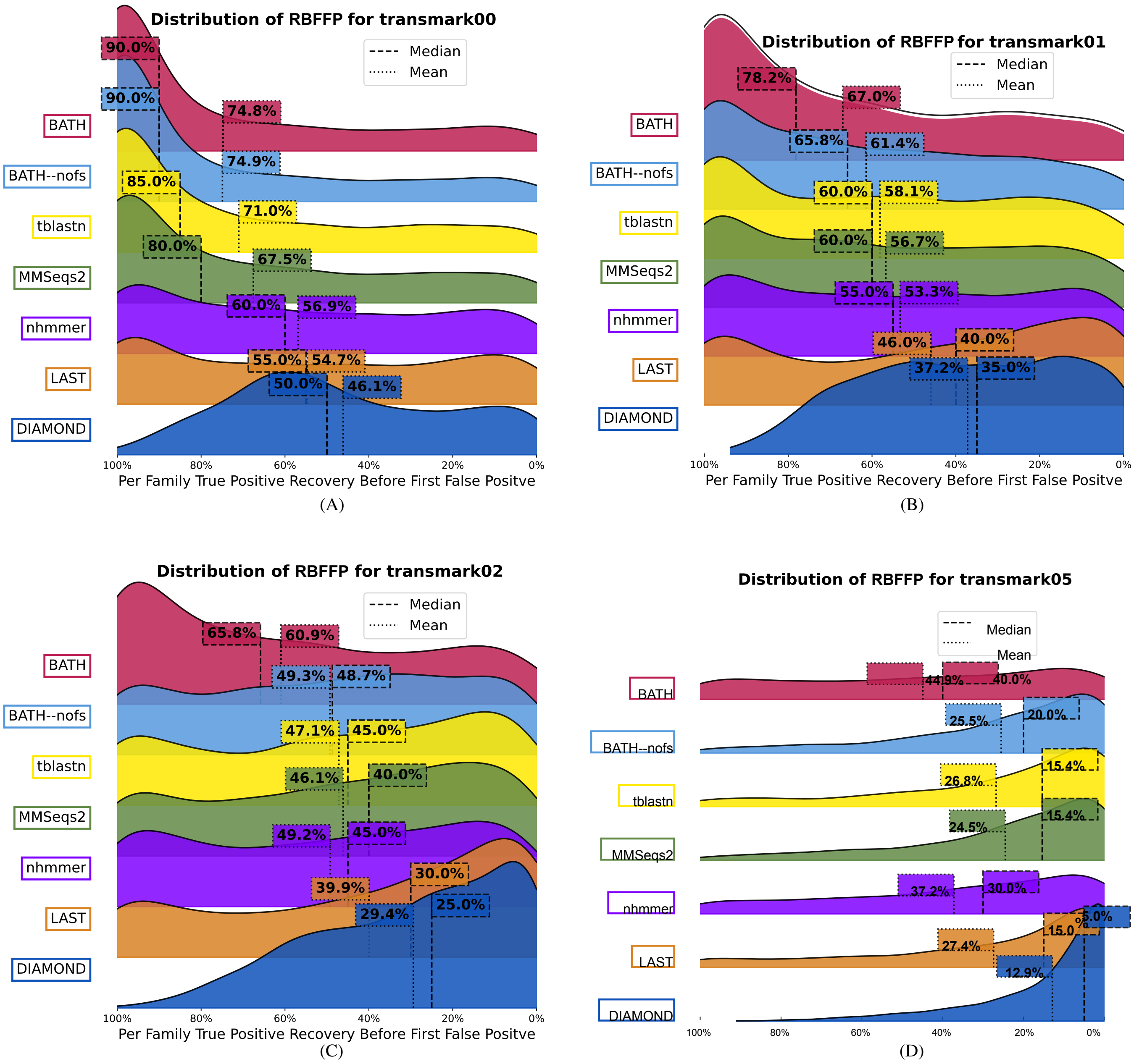

Fig. S3. Distribution of recall before first false positive (RBFFP) values across all query family searches, with various simulated frameshift rates. In the *transmark* benchmarks, protein-coding sequences belonging to 1,500 Pfam families (up to 20 instances per family) were embedded into ten 100MB simulated genomic sequences, along with 50,000 decoy open reading frames derived from shuffled Pfam sequences. For each family, up to 30 instances are kept as a query set (see Methods for details). A true positive is defined as a hit in which $> 50\%$ of the alignment is between a query from a family and an embedded test sequence from the same family. A false positive is defined as a hit where $> 50\%$ of the alignment is to either an embedded decoy or the simulated chromosome. Hits where $> 50\%$ of the alignment is between a query from one family and an embedded test sequence from a different family are ignored as it is not possible to discern whether true homology exists between any two families. To find the true positive recovery before the first false positive, the true and false positives from each tool were sorted by their reported E-values (smallest to largest). If a tool had more than one hit to the same test sequence only the hit with the lowest E-value is kept. For each family, RBFFP is computed as the fraction of true (embedded) positives fpr the family that were reported with an E-value lower than the first false positive. Ridgeline plots show the distribution of these per-family RBFFP values. (A) shows that BATH produces a greater fraction of families with high RBFFP on the non-frameshifted planted sequences than do other tools. The other three plots (B-D) show the decay in mean/median RBFFP as the frequency of frameshifts increases, and that BATH experiences less decay in coverage than other translated search tools as frameshift rates increase. All tests were performed using variant in which each family is represented by the full set of training family sequences, so that the query tool can either compute a profile or perform family pairwise search.
